# Rate of progressive healing with a carbon-fiber orthosis in cases of partial union and nonunion after ankle arthrodesis using the Ilizarov external fixator

**DOI:** 10.1186/s13047-023-00613-3

**Published:** 2023-03-25

**Authors:** Charlotte Cibura, Sebastian Lotzien, Thomas Rosteius, Christopher Ull, Periklis Godolias, Thomas Armin Schildhauer, Matthias Königshausen

**Affiliations:** Department of Trauma Surgery and General Surgery, BG University Hospital Bergmannsheil, Ruhr University Bochum, Bürkle-de-La-Camp-Platz 1, 44789 Bochum, Germany

**Keywords:** Carbon orthosis, Ankle arthrodesis, Ilizarov fixator, Nonunion, Partial union

## Abstract

**Background:**

The Ilizarov fixator is a popular device for treating arthrodesis of the ankle joint in complex situations. However, the therapy can fail, with nonunion or partial union that might not be load stable. There is the possibility of follow-up surgery or extended wearing of the fixator. Full weight bearing with a carbon orthosis remains another treatment option, which has not yet been investigated.The aim of the study was to determine the rate of progress that can be obtained with a carbon orthosis in cases of partial union or nonunion after fixator removal.

**Methods:**

In this retrospective observational study thirty-three patients received a carbon orthosis after fixator removal due to nonunion or partial union. All patients were allowed to walk with the orthosis under full load. The consolidation rate was determined radiologically and compared with the imaging data obtained during the last follow-up. In addition to demographic data, the Foot and Ankle Ability Measure and pain using a numeric rating scale were determined. Nine patients had to be excluded due to insufficient follow-up, and finally *n* = 24 patients were included in the study.

**Results:**

The average duration of fixator use was 21 weeks (range 15–40 weeks), and the total average follow-up after removal of the fixator was 16 months (range 4- 56). For 14 (58.33%) patients, there was a further increase in consolidation with the orthosis after the fixator was removed.

**Conclusion:**

The results show that if there is only partial union or nonunion, further consolidations can be achieved after the application of a carbon orthosis. In a difficult patient population, using an orthosis should therefore be attempted to avoid unnecessary revision operations.

## Background

For many years, good treatment results have been achieved with the Ilizarov fixator for arthrodesis in the ankle joint area. Ilizarov fixator application is an external procedure that has been described in particular for patients complicated with acute or chronic infections, soft tissue defects, axial malpositions (varus/valgus) and relevant comorbidities (diabetes mellitus [DM], polyneuropathy [PNP], peripheral vascular disease [PVD]). The healing results described range between 73 and 100% [1–13]. Most authors only describe the result of arthrodesis either as a union or as a failure in the sense of nonunion and/or infection. Only a few studies have described individual cases of partial consolidation or stable pseudarthrosis that were treated with an orthosis, and only in recent individual studies were the consolidation rates determined adequately. However, these were internal arthrodesis procedures, and there is no uniform consensus on when arthrodesis is likely to be stable [1, 4, 9, 10, 14–17]. In cases of nonunion or partial consolidation, there is the possibility of extending fixator application, follow-up surgery to perform cancellous bone apposition with or without a change of procedure to an internal procedure (plates/screws), rearthrodesis using an Ilizarov fixator or lower leg amputation [1, 4, 7–10]. Both rearthrodesis via the Ilizarov fixator and a process change to an internal procedure, however, can be made more difficult by the presence of vulnerable soft tissues, chronic osteomyelitis, multiple previous surgeries with already significant leg length differences, or incompliance or rejection by the patient because of repeated long and complex therapy. Additionally, a prolonged fixator wearing time is often not tolerated by the patient because of the high rates of complications such as recurrent pin infections or broken pins [1, 18]. The alternative to lower leg amputation in these cases can be the adaptation of a custom-made orthosis, provided there is no fulminant infection. The attempt to mobilize in an adapted orthosis in cases of partial union or nonunion after Ilizarov arthrodesis without any further internal osteosynthesis has only been described in very few cases, and so far, we are concerned that there are no studies on the further courses of these patients [1, 2, 4, 9, 10, 13, 19]. The aim of this study was therefore to determine clinical and radiological results in a difficult patient population with orthosis after fixator removal and existing nonunion or partial union. Can bony consolidation increase even after several months of wearing the fixator due to a full load in the orthosis? What clinical results can this procedure achieve? Could the orthosis be a possible sensible alternative to another revision or amputation in such a difficult patient population?

## Methods

### Study design

The present study was performed in accordance with the Declaration of Helsinki. Ethical permission for this study was obtained from the ethics committee and informed consent was obtained from all patients before participation in the study (registration number: 18–6582-BR). This is a retrospective register study with a prospective follow-up.

### Patient selection

All patients who underwent ankle fusion using the Ilizarov external fixator at our institution (level 1 trauma center) from 01/2013 to 01/2020 were retrospectively reviewed. All patients were included if they had tibiotalar joint (TT) arthrodesis, subtalar joint arthrodesis, simultaneous TT and subtalar joint arthrodesis or tibiocalcanear (TC) arthrodesis and had received a carbon orthosis after fixator removal due to lack of consolidation or partial consolidation that was (in our opinion) not guaranteed to be load stable without additional stabilization. For all these patients, further surgical treatment or extended fixator wearing was ruled out if they presented with vulnerable soft tissues/chronic osteomyelitis (COM)/previous illnesses/recurrent complications or if they refused such treatments. To capture all patients with these criteria, a keyword analysis of all digitized files was performed by the authors. The medical records of these patients were retrospectively reviewed for the following factors: sex, age, associated relevant concomitant diseases, body mass index (BMI), smoking, reason for arthrodesis, time spent in the fixator, follow-up, consolidation rate, consolidation rate at the last follow-up and wearing time of the orthosis. The data were collected anonymously using Microsoft Excel © Version 14.7.7. The exclusion criteria were as follows:

1. Follow-up less than six months from the start of Ilizarov fixator application
2. Patients treated with the Ilizarov fixator for bone transport or fracture treatment
3. Wearing time of the orthosis less than three months
4. Age < 18 years

### Incidence of fusion

The Incidence of fusion was determined according to the imaging results following fixator removal. It was divided into five groups (0%, 5–20%, 21–40%, 41–60%, 61–80%) and compared and evaluated with the imaging findings during the last follow-up examination. Fusion of a joint segment was defined as trabeculation or calcific density crossing the former space. In a total of 22 (91.67%) patients, computed tomography (CT) was performed at the time of fixator removal to determine consolidation. In 10 (41.67%) patients, this CT scan was compared with a scan obtained during the last follow-up. The consolidation rate was calculated based on the method by Jones et al. All sagittal images were retrospectively reviewed, the lengths of the fused segments and the lengths of each joint on each slice were recorded, and the incidence of fusion was then calculated: fusion incidence = 100x (sum of lengths of fused segments on all slices/sum of lengths of joint surface) [14].

For 12 (50%) patients, X-ray images + CT were compared with the X-ray images obtained at the last follow-up. For these patients, consolidation was also first determined using the abovementioned CT-based method, and then the course was determined using the X-ray images. For two (8.33%) patients, only X-rays were compared with regard to trabeculation or calcific density crossing the former space. Follow-up CT was often not performed due to a lack of symptoms, the detection of an already clear change on X-ray or the patient’s refusal of further treatment and thus ethically intolerable increased radiation exposure. All patients were in our regular treatment while the fixator was in place (presentation took place every 14 days), and the indication for fixator removal and the application of an orthosis was made by the authors in each case.

For the evaluation of this study, all images were finally evaluated independently by two investigators (orthopedic surgeons), and if there were any discrepancies, the images were rated by a third independent person.

### Treatment with the carbon orthosis

All patients included in this study received a custom-made lower leg carbon orthosis, which they were instructed to wear daily for mobilization for at least 3–6 months or longer if necessary. Full weight bearing was allowed with the orthosis.

**Fig. 1:**
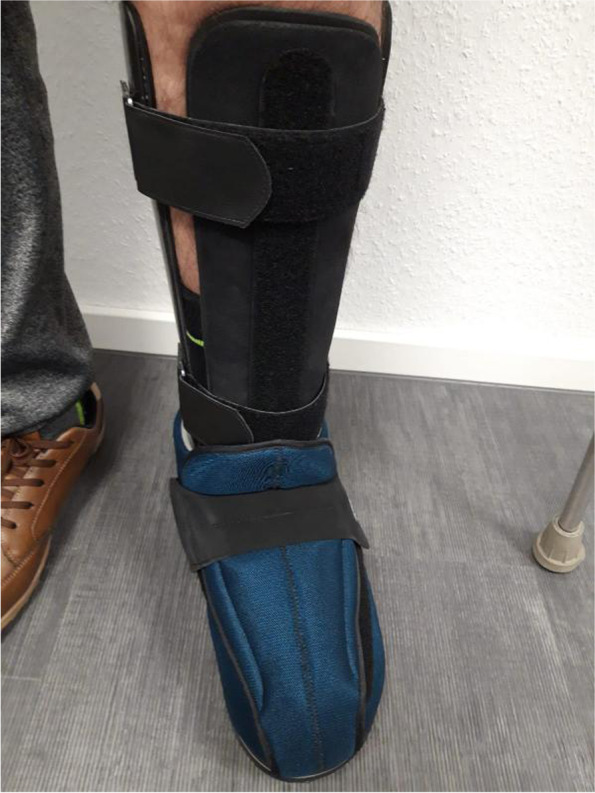
Picture of a custom-made carbon orthosis

**Fig. 2:**
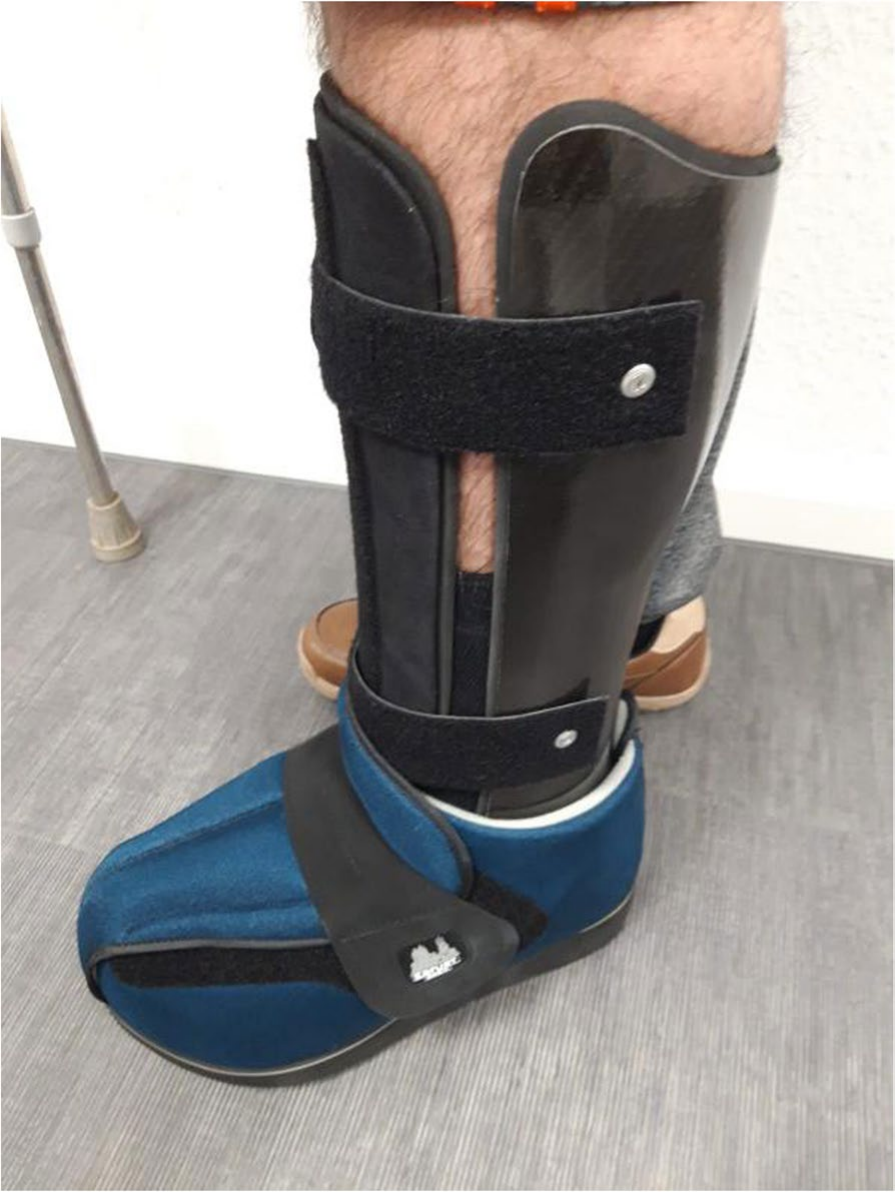
Picture of a custom-made carbon orthosis

**Fig. 3:**
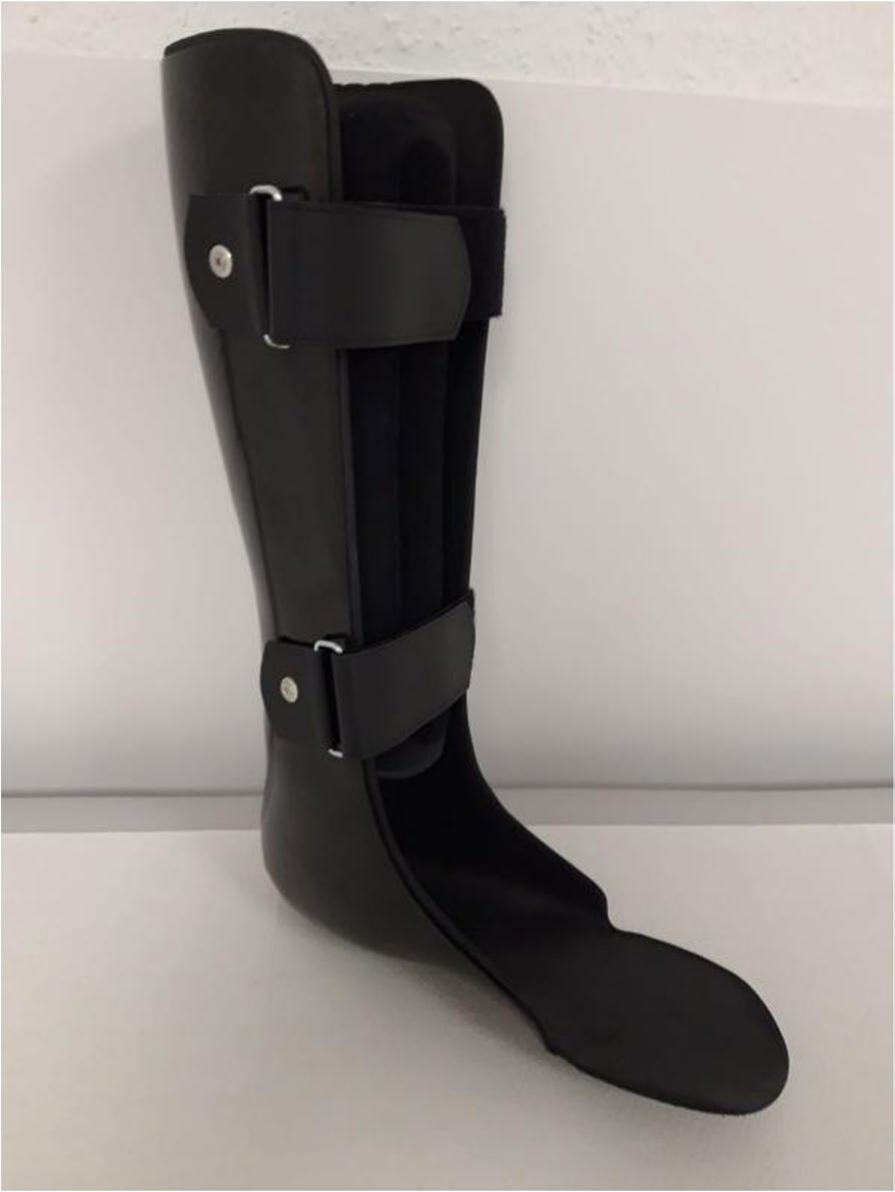
Picture of a custom-made carbon orthosis

Lower leg orthoses with permanently fixed ankle joints consist essentially of a lower leg shaft that is open towards the front, crosses the calves and extends proximally to about the knee bend, which merges distally into a foot section that is also open toward the front, reinforced in the ankle area and elastic in the forefoot area. The entire orthosis is mainly made of carbon fiber, with the exception of the elastic forefoot, which is made of aramid fiber. The orthosis is fixed to the lower leg with Velcro fasteners. The foot area is designed so that, typically, commercially available shoes can be worn, which in turn hold the foot/ankle area (Figs. 1, 2 and 3). The orthosis was made by the orthopedic and medical supply store Care Center Rhein Ruhr GmbH, Germany.

### Outcome score

To be able to determine pain and further outcomes, a prospective questionnaire with a numeric rating scale (NRS) and the validated German version of the Foot and Ankle Ability Measure (FAAM) was sent by mail and completed. The numeric rating scale was used to determine the actual pain, ranging from 0–10, with 0 representing no pain at all and 10 representing maximum pain. The FAAM questionnaire was used to determine the possibility of everyday and sporting activities for the patients [20]. The resulting score has previously been shown to be a reliable, valid and responsive outcome score of physical function in individuals with foot and ankle dysfunction [21]. The score comprises two subareas, one with 21 questions relating to everyday problems (activities of daily life [ADLs]) and a sports subarea with eight questions. The results of the individual questions are added and shown as a percentage. A maximum of 100% can be achieved in each case. Additionally, patients had to rate their functioning in ADLs and sports with a percentage ranging from 0 to 100 (0 indicating an inability to perform ADLs or sport activities) and had to provide information about the functional status of their ankles/feet using a four-point rating scale.

## Results

### Patients

In the specified period, 319 patients experienced arthrodesis in the area of the ankle using an Ilizarov fixator. For 33 (10.34%) of these patients, after removal of the fixator, a carbon orthosis was applied due to an existing nonunion or partial consolidation and the exclusion of further therapy options. Of these 33 patients, nine had to be excluded due to insufficient follow-up, and finally *n* = 24 (7.52%) patients (follow-up rate: 72.73%) were included in the study. Table 1 provides an overview of the patient group. The total average follow-up after removal of the fixator was 16 months (range 4- 56). The Ilizarov fixator was used externally for all patients because of the presence of an acute or chronic infection (17 [70.83%] cases) and/or poor soft tissue, secondary diseases or noncompliance.

**Table 1:** Study group *N* = 24

Study group	
**Average age (years)**	59 (range 27–77)
**Sex**	
Male	15 (62.5%)
Female	9 (37.5%)
**Duration in frame (weeks)**	21 (range 15–40)
**Average FU** **after fixator removal**	16 months (range 4- 56)
**TT arthrodesis**	14 (85.33%)
**Simultaneous TT/subtalar arthrodesis**	5 (20.83%)
**TC** **arthrodesis**	5 (20.83%)
**Rearthrodesis**	12 (50%)
**Average number of presurgeries**	6 (range 0–24)
**Comorbidities**	
Nicotine abuse	11 (45.83%)
DM	4 (16.67%)
Mean BMI	31.0 (range 21–41)
Wound healing disorders after fixator removal	3 (12,5%)
**Autogenous cancellous bone**	10 (41,67%)

*Abbreviations*: *TT*  tibiotalar, *TC*  tibiocalcanear, *FU*  follow up, *DM*  diabetes mellitus, *BMI* body measure index

### Incidence of Fusion

When evaluating the imaging findings in all cases, there was an agreement between the observers regarding an increase, decrease or no change in the consolidation. Different percentages were given for seven (29.17%) patients. In these cases, the images were also assessed by a third independent person, and a consensus could be found in all cases.

**Fig. 4:**
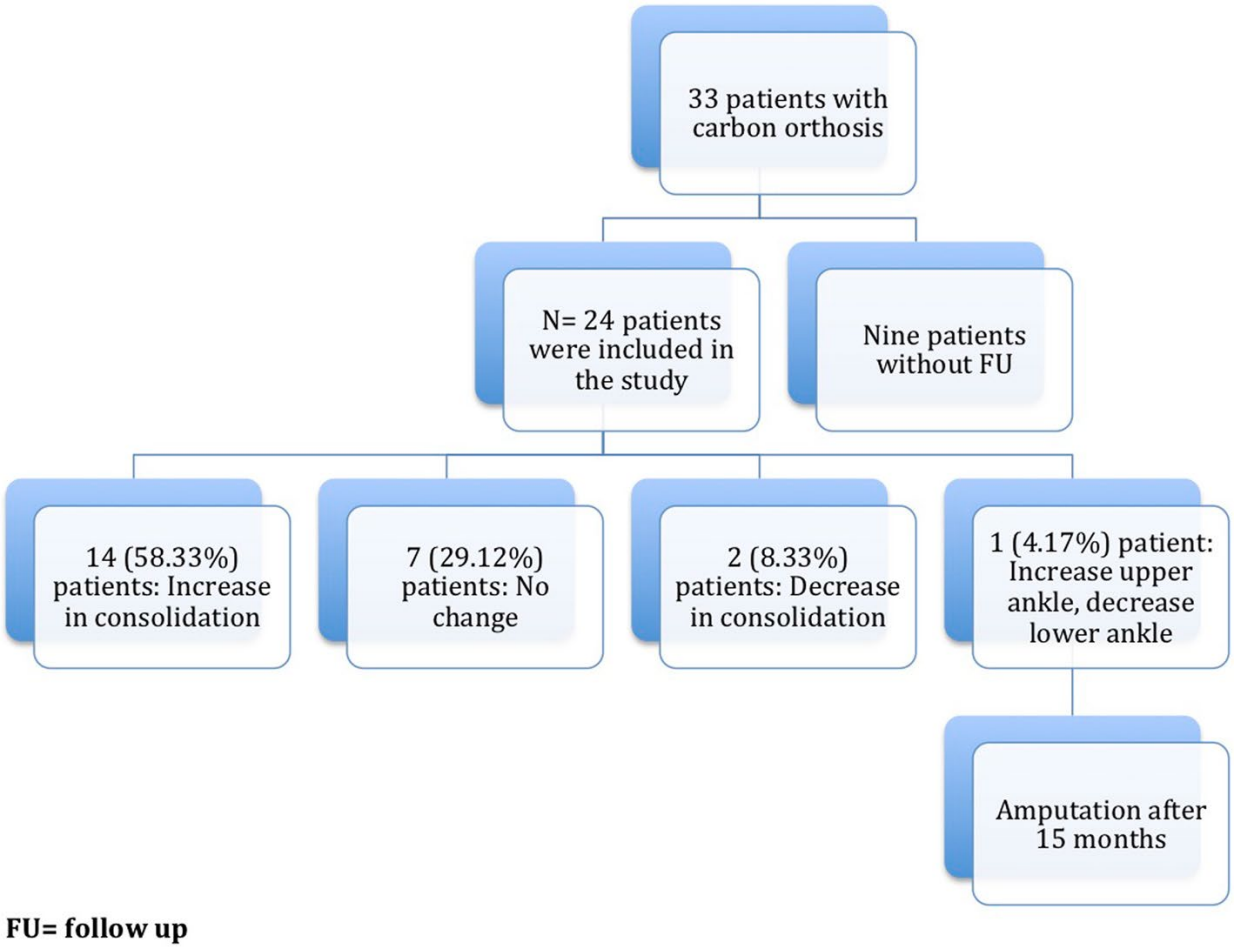
Overview of the courses in the orthosis after fixator removal

**Fig. 5:**
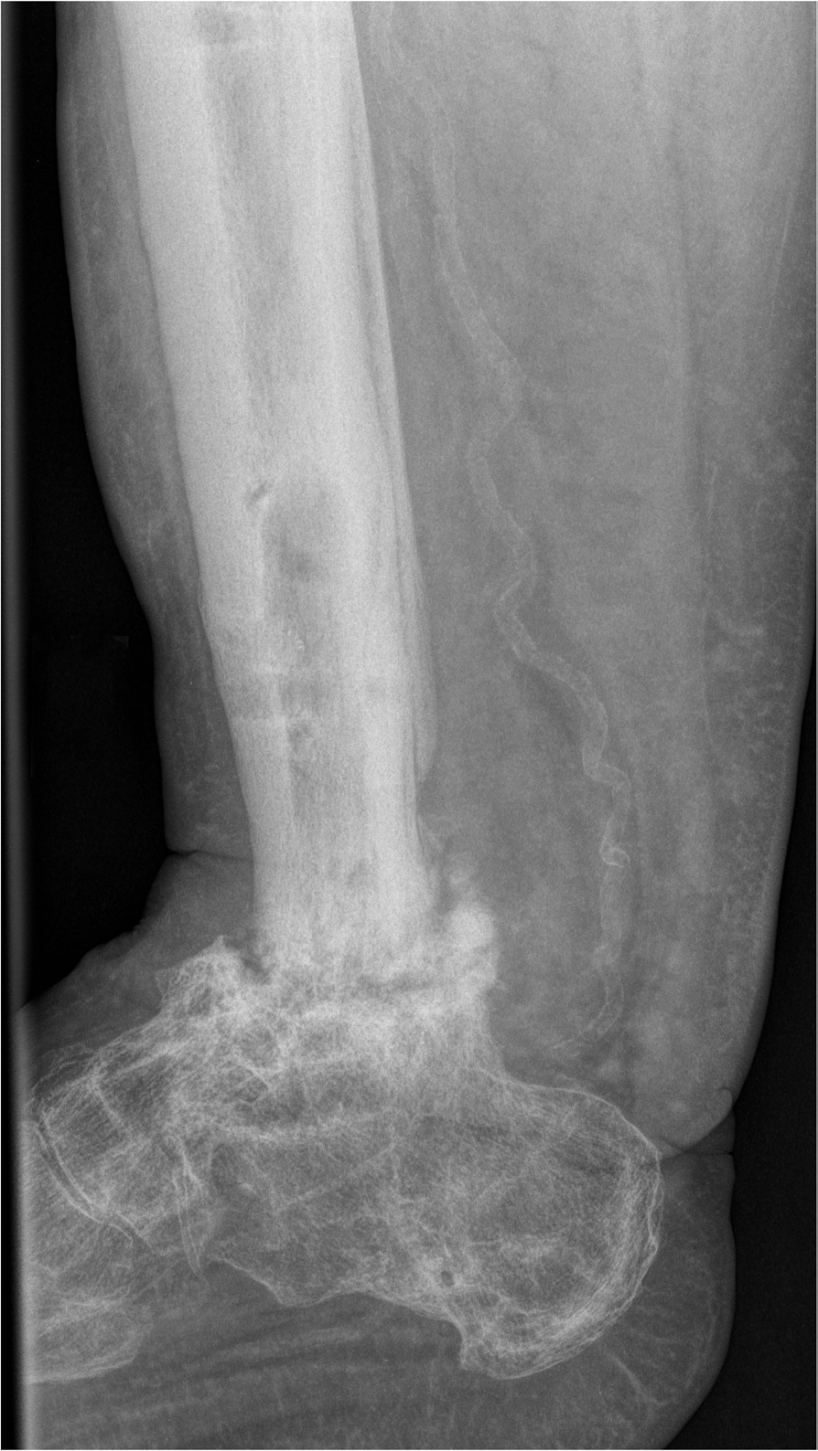
Case 1: X-ray (a.p. and lateral) and sagittal CT of a 57-year-old patient with a partial consolidation of 5–20% after fixator removal. The wearing time was 23 weeks. The initial ankle fracture was followed by multiple surgeries, leading to the development of chronic osteomyelitis and an already frustrating attempt at arthrodesis. In the last X-ray (a.p. and lateral), and after a follow-up of 19.5 months, there was a clear increase in consolidation. The patient stated that he had no pain. The FAAM score was 33 for the ADL part and 0 for the sports part. However, his BMI was 39.9, and the patient continued wearing the orthosis because it made him feel safer

**Fig. 6:**
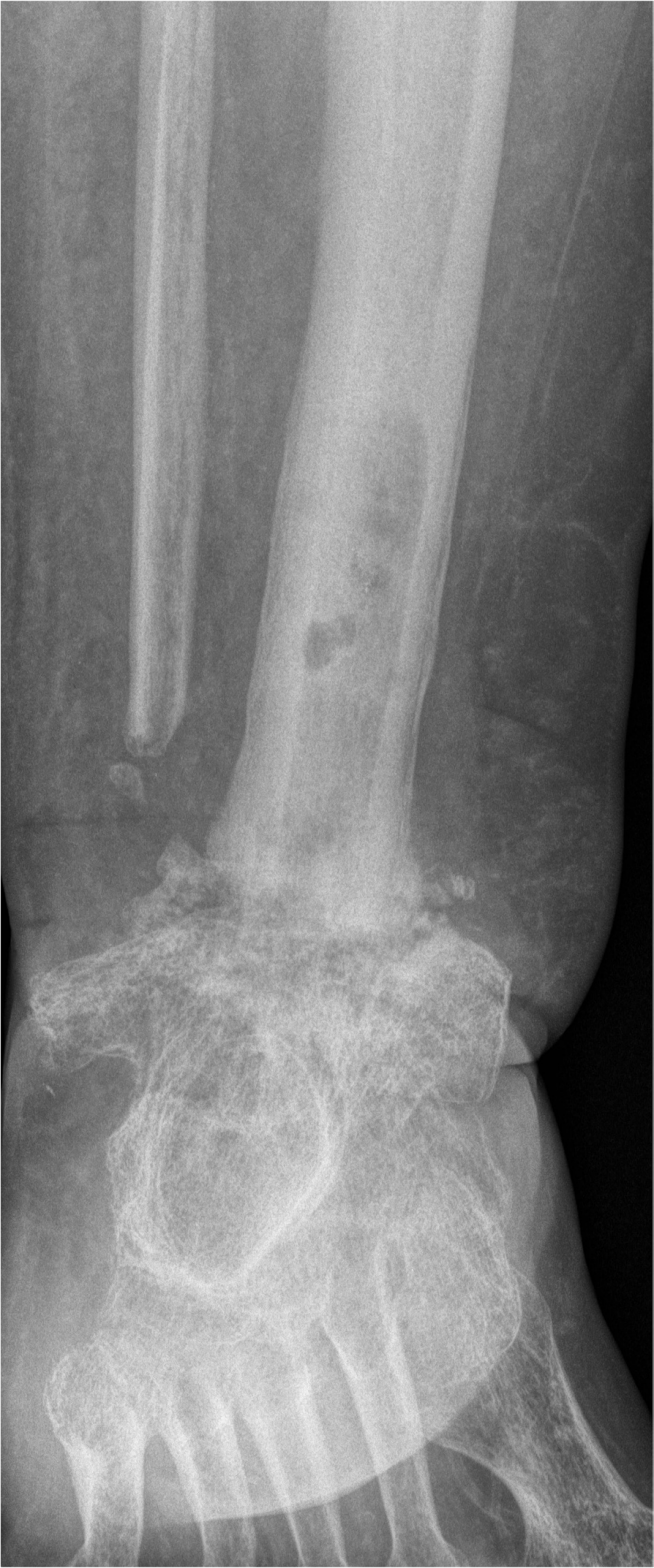
Case 1: X-ray (a.p. and lateral) and sagittal CT of a 57-year-old patient with a partial consolidation of 5–20% after fixator removal. The wearing time was 23 weeks. The initial ankle fracture was followed by multiple surgeries, leading to the development of chronic osteomyelitis and an already frustrating attempt at arthrodesis. In the last X-ray (a.p. and lateral), and after a follow-up of 19.5 months, there was a clear increase in consolidation. The patient stated that he had no pain. The FAAM score was 33 for the ADL part and 0 for the sports part. However, his BMI was 39.9, and the patient continued wearing the orthosis because it made him feel safer

**Fig. 7:**
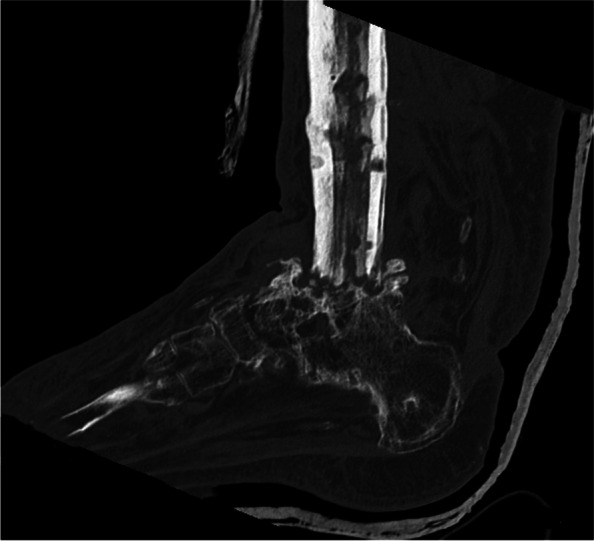
Case 1: X-ray (a.p. and lateral) and sagittal CT of a 57-year-old patient with a partial consolidation of 5–20% after fixator removal. The wearing time was 23 weeks. The initial ankle fracture was followed by multiple surgeries, leading to the development of chronic osteomyelitis and an already frustrating attempt at arthrodesis. In the last X-ray (a.p. and lateral), and after a follow-up of 19.5 months, there was a clear increase in consolidation. The patient stated that he had no pain. The FAAM score was 33 for the ADL part and 0 for the sports part. However, his BMI was 39.9, and the patient continued wearing the orthosis because it made him feel safer

**Fig. 8:**
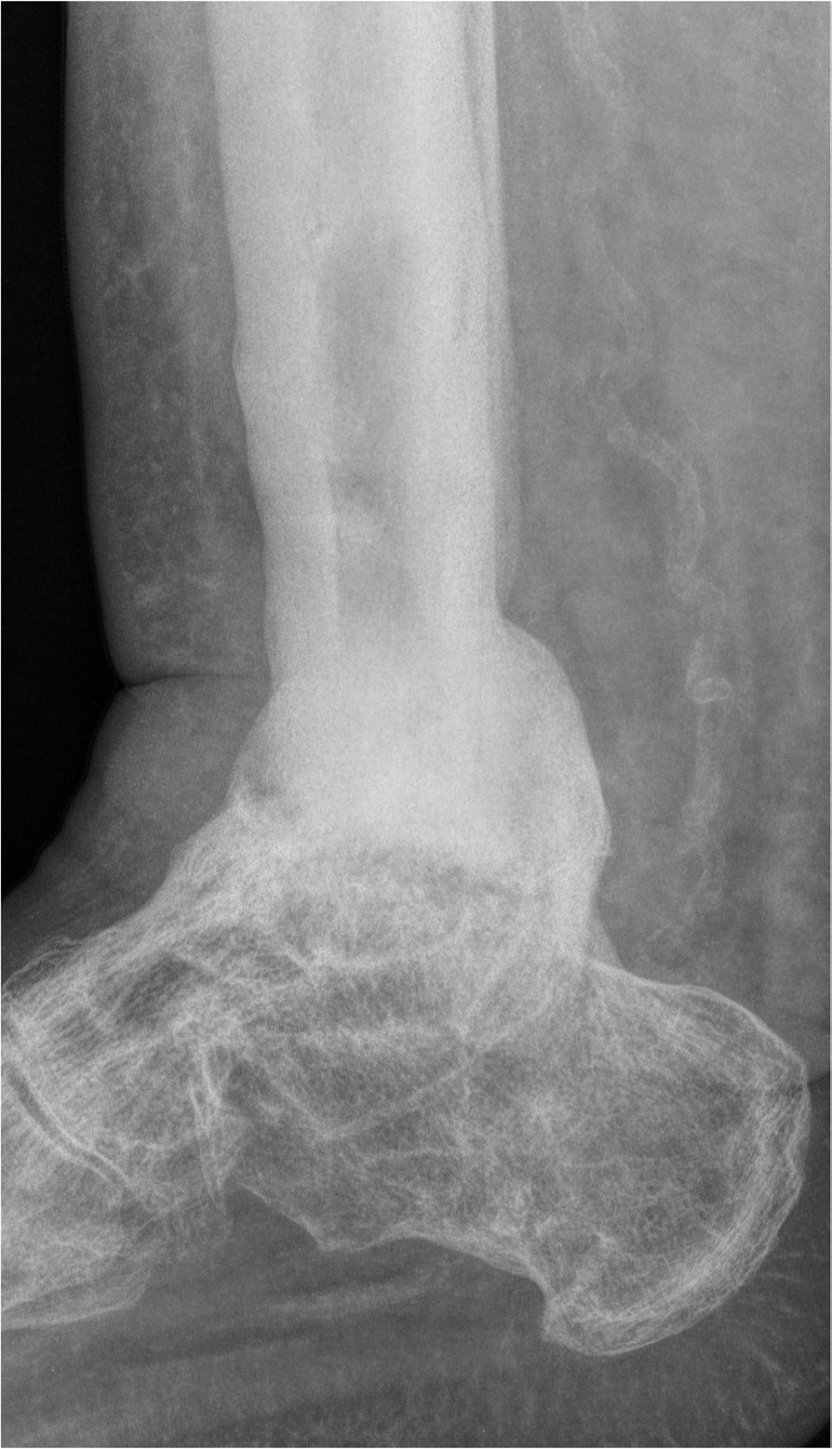
Case 1: X-ray (a.p. and lateral) and sagittal CT of a 57-year-old patient with a partial consolidation of 5–20% after fixator removal. The wearing time was 23 weeks. The initial ankle fracture was followed by multiple surgeries, leading to the development of chronic osteomyelitis and an already frustrating attempt at arthrodesis. In the last X-ray (a.p. and lateral), and after a follow-up of 19.5 months, there was a clear increase in consolidation. The patient stated that he had no pain. The FAAM score was 33 for the ADL part and 0 for the sports part. However, his BMI was 39.9, and the patient continued wearing the orthosis because it made him feel safer

**Fig. 9:**
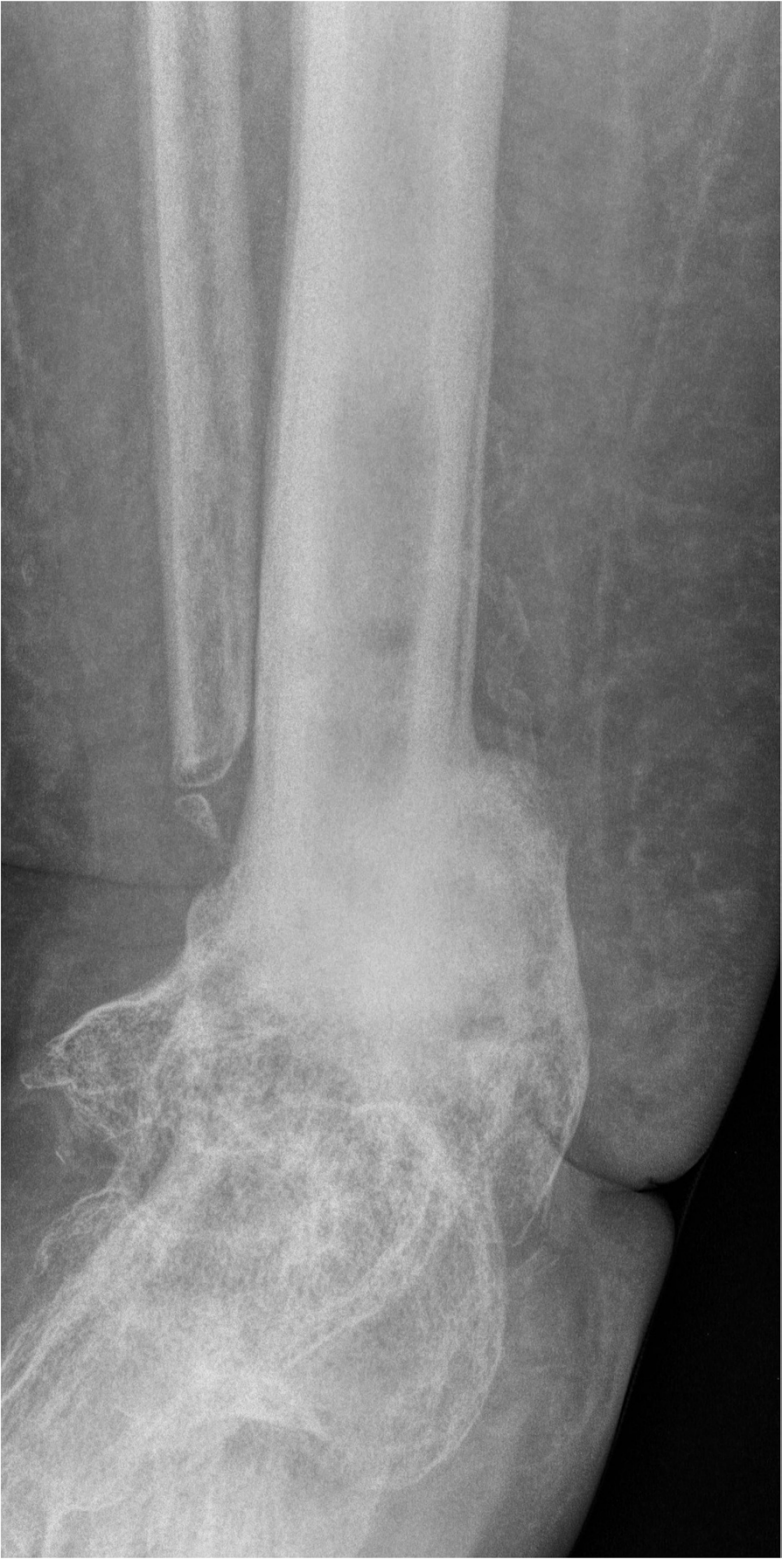
Case 1: X-ray (a.p. and lateral) and sagittal CT of a 57-year-old patient with a partial consolidation of 5–20% after fixator removal. The wearing time was 23 weeks. The initial ankle fracture was followed by multiple surgeries, leading to the development of chronic osteomyelitis and an already frustrating attempt at arthrodesis. In the last X-ray (a.p. and lateral), and after a follow-up of 19.5 months, there was a clear increase in consolidation. The patient stated that he had no pain. The FAAM score was 33 for the ADL part and 0 for the sports part. However, his BMI was 39.9, and the patient continued wearing the orthosis because it made him feel safer

**Fig. 10:**
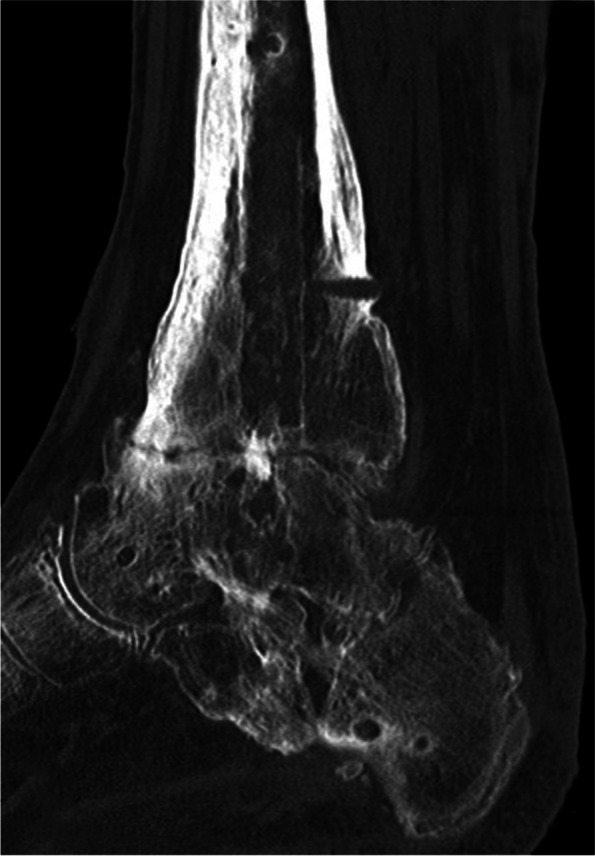
Case 2: CT (coronal and sagittal) of a 53-year-old patient with multiple surgeries and two frustrated attempts at arthrodesis using internal procedures. Four-month follow-up CT shows an increase in TT consolidation of up to 80–100%. The patient was able to train off the orthosis and switch to custom-made orthopedic shoes. The patient currently complains of pain with a visual analog scale score of 5 in the foot/ankle joint during exercise, but the CT now also shows clear arthrosis in the TN and CC joints

**Fig. 11:**
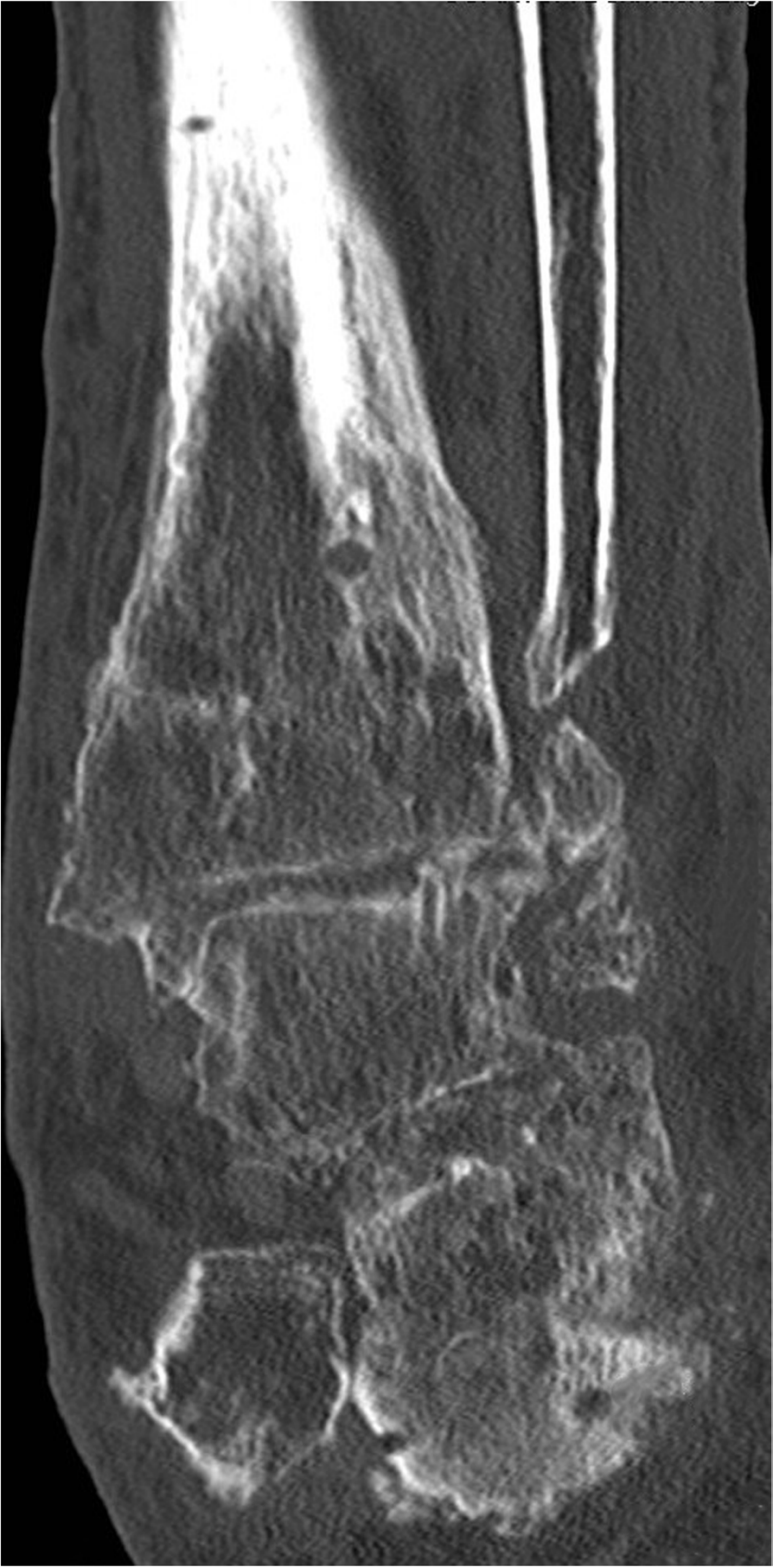
Case 2: CT (coronal and sagittal) of a 53-year-old patient with multiple surgeries and two frustrated attempts at arthrodesis using internal procedures. Four-month follow-up CT shows an increase in TT consolidation of up to 80–100%. The patient was able to train off the orthosis and switch to custom-made orthopedic shoes. The patient currently complains of pain with a visual analog scale score of 5 in the foot/ankle joint during exercise, but the CT now also shows clear arthrosis in the TN and CC joints

**Fig. 12:**
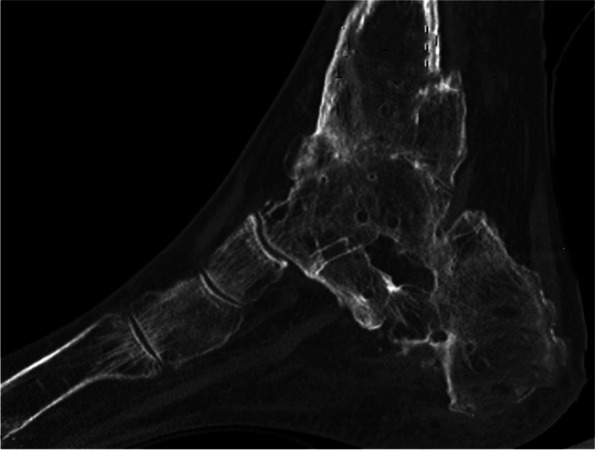
Case 2: CT (coronal and sagittal) of a 53-year-old patient with multiple surgeries and two frustrated attempts at arthrodesis using internal procedures. Four-month follow-up CT shows an increase in TT consolidation of up to 80–100%. The patient was able to train off the orthosis and switch to custom-made orthopedic shoes. The patient currently complains of pain with a visual analog scale score of 5 in the foot/ankle joint during exercise, but the CT now also shows clear arthrosis in the TN and CC joints

**Fig. 13:**
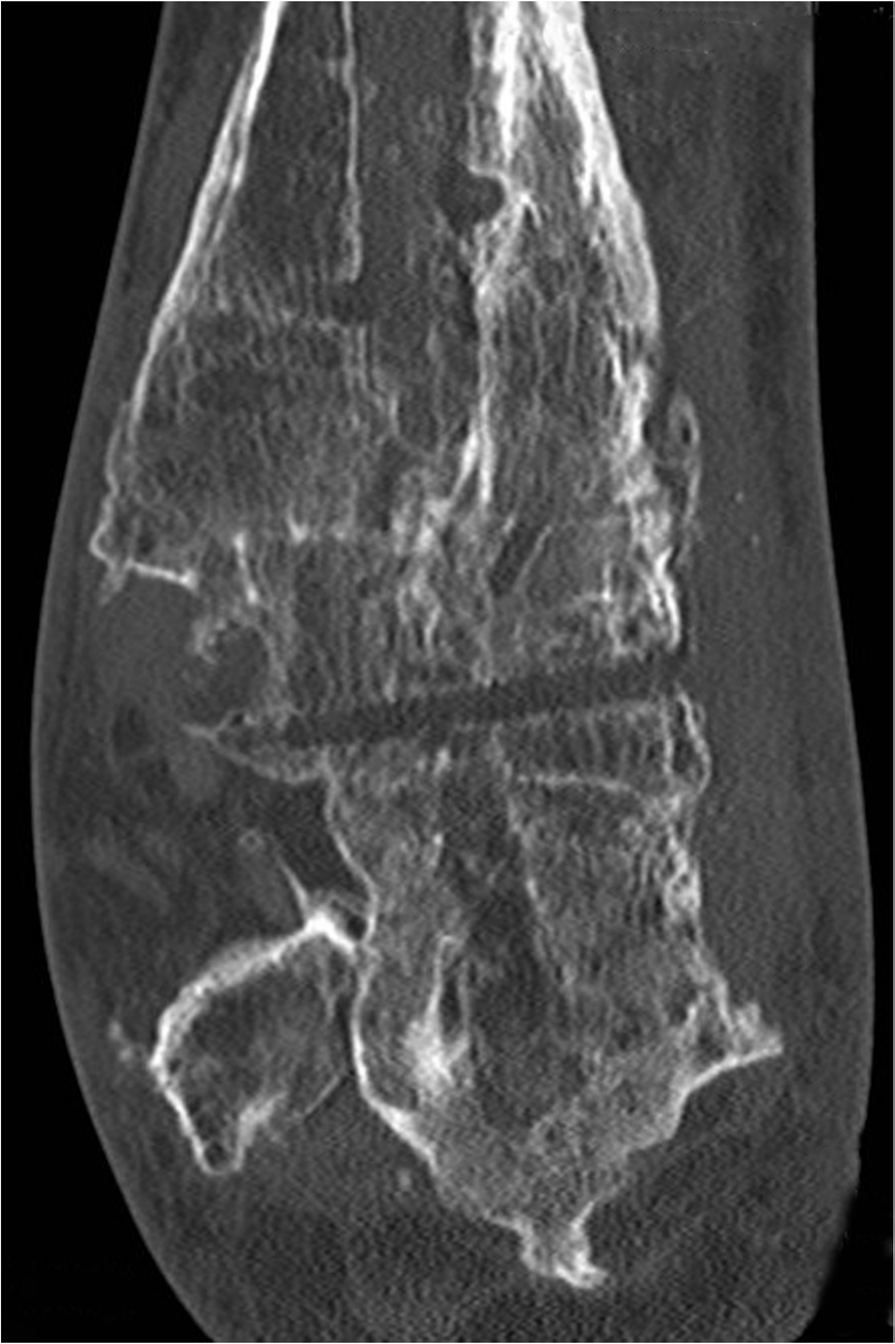
Case 2: CT (coronal and sagittal) of a 53-year-old patient with multiple surgeries and two frustrated attempts at arthrodesis using internal procedures. Four-month follow-up CT shows an increase in TT consolidation of up to 80–100%. The patient was able to train off the orthosis and switch to custom-made orthopedic shoes. The patient currently complains of pain with a visual analog scale score of 5 in the foot/ankle joint during exercise, but the CT now also shows clear arthrosis in the TN and CC joints

Figure 4 gives an overview of the course of the consolidation. For 14 (58.33%) patients, there was an increase in consolidation with the orthosis after the fixator was removed (Table 2); for seven (29.17%) patients, no change could be recorded (Table 3); for two (8.33%) patients, the consolidation decreased (Table 4); and for one (4.17%) patient, there was an increase in consolidation in the upper ankle and a decrease in consolidation in the lower ankle (simultaneous arthrodesis) (Table 5, Figs. 5, 6, 7, 8 and 9—Case 1, Figs. 10, 11, 12 and 13—Case 2). However, this last patient was amputated after 15 months due to an acute subtalar infection.

**Table 2:** Patient demographics and consolidation—14 (58.33%) patients with increase in consolidation (*N* = 24)

**ID**	**Age**	**Sex**	**BMI**	**Smoking**	**DM**	**Comorbidities**	**Diagnosis**	**Type of Arthrodesis**	**Consolidation at fixator removal in %**	**Consolidation at last FU in %**
6	68	m	30,7	No	Yes	HTN, asthma, alcohol abuse, PVD, sleep apnea, chronic kidney failure	Rearthrodesis, acute infection	TT + subtalar	TT 0, subtalar 0	TT 61–80, subtalar 61–80
19	57	m	39,9	No	No	none	Rearthrodesis, COM	TT	5–20	81–100
17	51	f	41,0	Yes	No	HTN,PNP, alcohol abuse	Post-traumatic arthrosis	TT	21–40	81–100
22	61	f	21,0	No	No	HTN, rheumatoid arthritis, total hip replacement + total knee replacement, wound healing disorder	Rearthrodesis, COM	TC	21–40	81–100
1	57	f	36,7	Yes	No	Rheumatoid arthritis, HTN, depression, scheuermann´s disease	Rearthrodesis, COM	TT	41–60	81–100
2	56	m	31,6	Yes	Yes	PNP, depression, neuropathic pain syndrome, wound healing disorder	Rearthrodesis, COM	TT	41–60	81–100
4	50	m	31,9	Yes	No	HTN, wound healing disorder	Post-traumatic, acute infection	TT	41–60	81–100
9	53	m	21,2	No	No	PNP	Re-rearthrodesis, post-traumatic arthrosis	TT	41–60	81–100
13	76	f	39,9	No	No	HTN,CAD,MI	Post-traumatic arthrosis	TT	41–60	81–100
20	77	m	21,9	No	No	HTN, MI, artrial fibrillation	Post-traumatic, acute infection	TT	41–60	81–100
21	60	m	36,0	No	No	HTN, thyroid cancer	Post-traumatic arthrosis	TT	41–60	81–100
23	67	f	28,4	No	No	Rheumatoid arthritis	Post-traumatic arthrosis	TT + subtalar	TT 41–60%, subtalar 81–100	TT 81–100, subtlar 81–100
5	58	f	20,8	Yes	No	none	Rearthrodesis, COM	TT	61–80	81–100
11	27	m	29,1	Yes	No	Hypothyroidism	Rearthrodesis, COM	TT	41–60	61–80

**Table 3:** Patient demographics and consolidation—seven (29.17%) patients with no change in consolidation (*N* = 24)

**ID**	**Age**	**Sex**	**BMI**	**Smoking**	**DM**	**Comorbidities**	**Diagnosis**	**Type of Arthrodesis**	**Consolidation at fixator removal in %**	**Consolidation at last FU in %**
3	51	f	32,6	No	Yes	Asthma	Acute infection	TT	61–80	61–80
7	64	m	22,2	Yes	No	HTN, PVD	Rearthrodesis, COM	TT + subtalar	TT 81–100, Subtalar 61–80	TT 81–100, subtalar 61–80
8	54	m	32,1	Yes	No	HTN, wound healing disorder	Posttraumatic arthrosis	TT + subtalar	TT 81–100, subtalar 5–20	TT 81–100, subtalat 5–20
12	48	m	31,0	Yes	No	HTN, lower leg amputation opposite	Rearthrodesis, COM	TC	0	0
14	60	m	26,3	No	No	none	COM	TC	5–20	5–20
15	52	m	38,0	Yes	No	HTN, asthma, chronic pain syndrome, tumor prosthesis hip	Rearthrodesis, idiopathic necrosis of the talus	TC	5–20	5–20
24	75	f	24,5	No	No	HTN, PNP	Rearthrodesis, acute infection	TC	0	0

**Table 4:** Patient demographics and consolidation—consolidation decreased in two (8.33%) patients (*N* = 24)

**ID**	**Age**	**Sex**	**BMI**	**Smoking**	**DM**	**Comorbidities**	**Diagnosis**	**Type of Arthrodesis**	**Consolidation at fixator removal in %**	**Consolidation at last FU in %**
10	66	m	38,8	No	No	HTN, artrial fibrillation, alcohol abuse, varicosis	Post-traumatic, acute infection	TT	61–80	5–20
18	60	m	38,6	No	Yes	Varicosis, chronic pain syndrome	Post-traumatic, COM	TT	21–40	0

**Table 5:** Patient demographics and consolidation—one (4.17%) patient with an increase in consolidation in the upper ankle and a decrease in the lower ankle (*N* = 24)

**ID**	**Age**	**Sex**	**BMI**	**Smoking**	**DM**	**Comorbidities**	**Diagnosis**	**Type of Arthrodesis**	**Consolidation at fixator removal in %**	**Consolidation at last FU in %**
16	60	f	32	Yes	No	Rheumatoid arthritis, PNP, COPD, osteoporosis, depression	Post-traumatic, Acute infection	TT + subtalar	TT 61–80, subtalar 61–80	TT 81–100, subtalar 21–40

*Abbreviations*: *F *female, *M* male, *COM* chronic osteomyelitis, *DM* diabetes mellitus, *PNP* polyneuropathy, *PVD* peripheral vascular disease, *HTN* hypertension, *CAD* coronary artery disease, *MI* myocardial infarction, *COPD* chronic obstructive lung disease, *TT* tibiotalar, *TC* tibiocalcanear

Of the 14 patients with an increase in consolidation, one (7.14%) patient initially had a consolidation rate of 61–80%, nine (64.29%) had a rate of 41–60%, two (14.29%) had a rate of 21–40%, one (7.14%) had a rate of 5–20% and one (7.14%) had a rate of 0%. In 12 (85.71%) of the 14 patients, there was 81–100% consolidation during an average follow-up of 18 months (range 4—56) (Table 2).

Of the seven patients without change, there was a consolidation of 0% among two (28.57%), 5–20% among three (42.86%) and 61–80% among two (28.57%) (Table 3). Here, the average follow-up was 11 months (range 4–33).

In two patients, the rate was between 21–40% and 61–80% after removal of the fixator and continued to decrease over the course to 0% and 5–20%. Here, the follow-up was seven and 46 months, respectively (Table 4).

### Treatment with the carbon orthosis

All patients wore the orthosis regularly during the daytime and for any kind of mobilization for at least 3 months. Sixteen (66.67%) patients were able to train off the orthosis after an average of 10 months (range 3–36) and switch to custom-made orthopedic shoes. Of these, nine patients had a consolidation of 80–100% in the last FU, two of 60–80%, one of 40–60%, two of 5–20%, and one of 0%, while one had no change (80–100% tibiotalar, 5–20% subtalar) in the last FU. Seven (29.17%) patients continue to wear the orthosis during mobilization today. Of these, two and one patient had 0% and 5–20% consolidation, respectively (all with clinical instability), one patient had 60–80% consolidation and three patients had 80–100% consolidation in the last FU. One further patient was amputated.

### Outcome score

The NRS and FAAM scores were fully recorded for 16 of 23 (69.57%) patients after an average of 3,2 years (after fixator removal). Seven (43.75%) patients stated that they had no pain at all. Of these, three patients had a consolidation of 81–100% at the last radiological FU, one patient had a consolidation of 61–80%, one had 81–100% TT and 61–80% subtalar consolidation, one patient had 41–60% consolidation, and one had a consolidation of 5–20%. The remaining nine (56.25%) patients reported pain in the ankle, which averaged 4,8 on the numeric rating scale (range 2–8). Of these, at the last FU, two patients had 0%, one had 5–20%, one had 61–80%, four had 81–100% and one had 81–100% tibiotalar and 0–20% subtalar consolidation. However, three of these last six patients already had advanced arthrosis in the subtalar or calcaneocuboid joint (CC) and talonavicular joints (TN), and one patient already had consolidation of the CC and TN during the last radiologic FU.

The FAAM score showed an average value of 49% (SD ± 18) on the subscale for ADL and 24% (SD ± 17) on the sports subscale. The results of the FAAM score along with a listing of the individual subscales are shown in Table 6.

**Table 6:** FAAM score for 16 patients (ADLs and Sports)

**FAAM, mean ± SD, %**	**Value**
**ADL subscale**	49 ± 18
**ADL global 0–100 rating scale**	38 ± 20
**Sport subscale**	24 ± 17
**Sport global 0–100 rating scale**	21 ± 26
Higher scores represent higher levels of function for each subscale, with 100% representing no dysfunction	
**FAAM overall level of function in %**	
**Normal**	0
**Nearly normal**	18
**Abnormal**	47
**Severely abnormal**	35

## Discussion

In the difficult patient population presented in this study, after fixator removal and under full load in a carbon orthosis, there was an increase in the bony consolidation of the arthrodesis zone in 58.33% (*n* = 14) of all 24 cases. This took place after the fixator had already been worn for an average of five months, and further risky surgeries were thus avoided. In most cases, there was consolidation between 41–60% after fixator removal, but an increase could also be recorded in patients with 21–40%, 5–20% and 0% consolidation. Mobilization under full load was possible for all patients.

In complex patient populations such as that described in this study, with multiple previous surgeries, already failed arthrodesis attempts, an infection history, poor soft tissues, a long medical history and/or relevant previous illnesses, the decision to repeat surgical treatment in cases of nonunion or partial union after wearing the Ilizarov fixator for several months is often difficult. In our opinion, the risk of a repeated infection following introduction of internal osteosynthesis material would have been too high for many of these patients, and/or the soft tissue situation and previous illnesses would have meant an increased risk of a defect/wound healing disorder. An extension of the wearing time of the fixator or even another attempt at arthrodesis using the Ilizarov fixator is often not tolerated by the patient due to its bulky structure and the possibility of recurrent pin infections, further complications, injuries to the opposite leg and destruction of clothing and bed linen. The alternative is amputation. To prevent this, a carbon orthosis was first applied in this patient population with the provision of full weight bearing. To the best of our knowledge, the possibility of further treatment using a carbon orthosis in the case of partial consolidation or nonunion has not yet been described in cases of arthrodesis via an Ilizarov fixator.

In recent studies, primarily good results have been achieved with the Ilizarov fixator [1–13, 18]. However, most of these studies only described a union or a failure in cases of nonunion or infection. Arthrodesis is considered permanent if it is clinically stable and if X-ray or CT shows bridges, although this is described differently depending on the author. Khanfour et al. described arthrodesis as the detection of bridging trabeculation in at least 2 planes at the arthrodesis site on the radiogram [8]. Katensis et al. required evidence of bridging trabeculae without a change in the position of the ankle under weight bearing, and Salem et al. confirmed successful arthrodesis by painless weight bearing as well as radiologically using plain radiographs or CT scans [7, 18]. Since X-ray was not always sufficiently meaningful in the complex courses of the patients in the current study, CT was also performed for almost all patients after the fixator was removed. A much more precise statement about the consolidation of the arthrodesis could thus be made [14–17]. The abovementioned patients all showed only partial consolidation or nonunion. To avoid breaking the existing bone bridges in this complicated patient group and to achieve a further increase in consolidation even without a fixator or additional internal osteosynthesis material, an orthosis was applied. This allowed all patients to walk, and repeated surgical treatment with all risks and complications was avoided.

The grouping of the consolidation rate as sets of percentages was based on Jones et al. [14]. He described a system for calculating the extent of consolidation and divided it into the following groups: 0–33%, nonunion, 34–66%, partial consolidation, and 67–100%, consolidation. However, this was via the application of an internal arthrodesis procedure. Further studies by Dorsey et al. and Glazebrook et al. followed this system for internal arthrodesis as well and stated that arthrodesis is stable from over 33% or over 25- 49% [15, 17]. For internal arthrodesis, there is therefore no consensus regarding the consolidation rate on CT when partial consolidation is sufficiently stable. After external arthrodesis using an Ilizarov fixator, very few authors describe partial consolidations or tight pseudarthroses that were subsequently treated with an orthosis. However, the further course of the patients remains unclear. Kugan et al. described a patient with nonunion who could mobilize with an orthosis with pain. El Alfy et al. also described a patient with fibrous nonunion who was able to mobilize with an orthosis, and Zarutzy et al. described five patients who needed a supporting orthosis (four stable pseudarthroses and one malunion) [4, 9, 10]. In a study on tibiocalcanear arthrodesis by Reinke et al., a patient with a partial consolidation of 40–50% and four patients with stable pseudarthrosis were described, all of whom were also treated with an orthosis [1].

In this study, 14 (58.33%) patients under load in the orthosis showed an increase in consolidation six months after the start of arthrodesis. In nine (64.29%) patients, there was an initial consolidation of 41–60%, and in some cases, an orthosis might not have been necessary. However, given the long course of the disease, we did not want to take any risks, and the orthosis initially provides the patient with security so that a full load is possible. In four other patients, the consolidation was 0% (1), 5–20% (1) and 21–40% (2). The risk of a break in areas with few bridges would have been too high, and a safe increase could be achieved with the orthosis. Among the seven (29.17%) patients with no change, the majority only had 0% (2) or 5–20% consolidation (3), which could indicate that an increase occurs sooner when at least 21–40% ossification has already occurred. However, the follow-up for these patients was shorter at 11 months (average) than for the patients with an increase (17.4 months), and with such a small number, no definitive statement can be made.

The 58.33% of the patients who experienced an increase in consolidation does not seem very high at first, but this must be considered against the background of a difficult patient population. Eleven (45.83%) patients were smokers, the average BMI of 31.0 was in the range of obesity grade 1, four (16.67%) patients had diabetes mellitus, three (12.5%) patients presented with wound healing disorders during as well as after fixator removal, and other difficult diseases such as chronic kidney failure with renal osteopenia, osteoporosis, rheumatoid arthritis, PVD, depression, and alcohol abuse were recorded (Tables 2, 3, 4 and 5). Twelve (50%) patients experienced a second attempt after a previous frustrating arthrodesis, and one patient experienced a third attempt. In a study on 88 patients with internal subtalar arthrodesis, Chahal et al. showed that smokers and patients with DM had a 3.8- and 18.7-fold higher probability of malunion and that the worst functional outcome was observed in patients with DM [22].

The results of the FAAM score were significantly worse for the ADL subscale with 49% (SD ± 18) and for the sport subscale with 24% (SD ± 17) than for other studies. Kerkhoff et al. described an average value of 70% (SD ± 22.3) for the ADL subscale and 29% (SD ± 27.8) for the sport subscale in 122 patients. However, these were primary internal arthrodeses in the case of degenerative changes, and the BMI was lower at 27.5 (SD ± 4,9); no statement was made regarding DM [23]. Strasser et al. described a score of 81.5 (SD ± 18.3) in patients over 70 after internal arthrodesis [24]. The BMI or other previous illnesses were not reported. A division into subscales was not made here either. Morasiewicz et al. achieved values of 79% (56–88) after Ilizarov arthrodesis and 70% (49–91) after internal arthrodesis [25]. Although this included patients with infection, vulnerable soft tissues and deformities, there are no reports of complicated courses with frustrating arthrodesis in advance. The average age of 44 years was also significantly younger than that of 59 years in this study. However, it shows that the overall outcome with partial consolidation and orthosis for complicated patients (multiple interventions, frustrating previous arthrodesis attempts, long disease courses and/or multiple previous illnesses) is worse overall, so orthosis should be seen as a salvage procedure and only be provided an alternative for individual cases.

### Limitations

This study has several limitations. The study sample of 24 patients was small, and the study mainly had a retrospective design. However, these are complex individual patients after following Ilizarov arthrodesis, which is otherwise a well-investigated therapeutic procedure; therefore, it is not expected that a large number of patients should be encountered. Even if CT had been performed for almost all patients after fixator removal, only 10 (41.67%) patients underwent follow-up CT. In the remaining patients, CT and X-rays were compared with only X-rays. However, further CT diagnostics would in some cases not have been ethically justifiable if the change was already clearly visible on comparable X-ray images or because there would have been no further consequences for the patient. Thus, the radiological percentages could not be determined exactly.

For patients without a change, the follow-up was significantly shorter than for patients who did demonstrate change, so no statement can be made here as to whether a change might still occur in the course of the process, which would potentially increase the rate of ossification even further. The outcome scores could not be recorded for all patients because some had moved to an unknown location or had already died from other causes. A longer follow-up would be necessary here to make a reliable statement regarding the long-term outcome. In addition, there was no comparable control group without an orthotic device, so no statement can be made regarding the course in this case.

## Conclusion

In conclusion we found that in cases of difficult patients or tissue conditions and partial consolidation after arthrodesis in the ankle joint using the Ilizarov fixator, further ossification can be detected in some patients after fixator removal following the use of a carbon orthosis. Thus, further surgical treatment or extended wearing of the fixator could be avoided in such complex cases.

## Data Availability

All of the data are electronically available.

## Abbreviations

ADL: Activities of daily life
FAAM: Ankle Ability Measure
BMI: Body mass index
CC: Calcaneocuboid joint
COM: Chronic osteomyelitis
CT: Computed tomography
DM: Diabetes mellitus
FU: Follow up
NRS: Numeric rating scale
PNP: Polyneuropathy
PVD: Peripheral vascular disease
TT: Tibiotalar
TC: Tibiocalcanear
TN: Talonavicular joints
